# Shrinkage Crack Patterns of Rectangular Timber Beams and Their Influence on Load-Bearing Capacity

**DOI:** 10.3390/ma19050942

**Published:** 2026-02-28

**Authors:** Xiaoyi Hu, Jiawei Wu, Xuwei He, Lu Li, Wei Guo, Jingjing Yang

**Affiliations:** 1 College of Optical, Mechanical and Electrical Engineering, Zhejiang A&F University, Hangzhou 311300, China; wujiawei@stu.zafu.edu.cn (J.W.); 2025612021008@stu.zafu.edu.cn (X.H.); ekoi@stu.zafu.edu.cn (W.G.); kilashoo@stu.zafu.edu.cn (J.Y.); 2 College of Chemistry and Materials Engineering, Zhejiang A&F University, Hangzhou 311300, China; lilu@zafu.edu.cn

**Keywords:** timber beam, shrinkage crack, crack pattern, load-bearing capacity, safety evaluation

## Abstract

**Highlights:**

**What are the main findings?**

- When multiple cracks exist in a timber beam, their collective effect is not a simple superposition of individual cracks but a spatial distribution coupling effect;
- In beams with multiple cracks, symmetric crack patterns result in a slightly lower decline in load-bearing capacity than asymmetric or single-crack patterns;
- Even deep shrinkage cracks do not lead to a complete loss of capacity.

**What are the implications of the main findings?**

- The non-linear spatial coupling effect of multiple cracks implies that the assessment and repair of cracked timber beams must account for the overall crack pattern and interaction, rather than simply evaluating or summing the influence of individual cracks in isolation;
- The repair and reinforcement of cracked beams should consider the overall crack pattern, not just the deepest crack, since symmetric or multiple interacting cracks can alter the stress distribution and the overall degradation of structural performance;
- Even deeply cracked beams retain significant residual capacity, indicating that historical timber structures with shrinkage cracks may not require immediate replacement, allowing for more targeted and economical repair strategies.

**Abstract:**

This study used finite element simulation and theoretical analysis to predict the crack distribution patterns that may occur during the shrinkage cracking process of rectangular timber beams. Based on the predictions, experimental specimens with six typical crack distribution patterns (I–VI) were designed. Subsequently, a four-point bending test method was employed to conduct large-sample size fracture tests on a total of 1200 small-sized *Pinus sylvestris* var. *mongolica* specimens, quantifying the effects of the crack depth, location, and distribution patterns on the specimens’ load-bearing capacity. The results indicate that when multiple cracks exist in a timber beam, their collective effect is not a simple superposition of individual cracks but a spatial distribution coupling effect. Both the depth and location of the cracks play crucial roles in their interaction. This study introduces three coefficients for evaluating the influence of cracks on timber beams, namely the load-bearing capacity coefficient (*R*), the decline ratio of load-bearing capacity (*D*), and the comprehensive crack-influence coefficient (*β*), which can effectively quantitatively evaluate crack damage effects. The framework established in this study, which links shrinkage crack characteristics with the load-bearing capacity of timber beams, along with the experimental data provided, can serve as a reference for the safety evaluation and scientific maintenance of historical timber components and modern timber structures with shrinkage cracks.

## 1. Introduction

As a green, renewable building material with excellent mechanical properties, wood has played a pivotal role in architectural structures across the world, in both ancient and modern times [1]. However, as a biomass material, wood exhibits strong hygroscopicity, with its dimensions and mechanical properties undergoing significant changes in response to variations in environmental humidity [2]. During the drying process of wood, non-uniform moisture content gradients within the material can induce drying stresses. Once these stresses exceed the transverse tensile strength of the wood, shrinkage cracks may form on the surface [3,4]. Such cracks represent one of the most common forms of damage in timber beams and can significantly compromise their load-bearing capacity [5].

To assess the potential impact of shrinkage cracks on timber beams, it is essential to first understand the formation patterns of these cracks. Theoretical models and numerical simulations serve as powerful tools for gaining an in-depth understanding of this complex physical process. For instance, Peng et al. found that in timber beams with a height greater than their width, there is a significant difference in the depth of shrinkage cracks between the side surfaces and the top/bottom surfaces (usually, the cracks on the top and bottom surfaces are deeper). Carlos et al. simulated the moisture transfer and stress–strain behavior of eucalyptus during isothermal drying, revealing the stress development mechanism induced by moisture content gradients [6]. Florian et al. simulated the drying and cracking process of wooden components under indoor climate conditions, establishing a quantitative relationship between humidity gradients and maximum crack depth, and demonstrating that the initial moisture content plays a decisive role in crack development [7]. Hu et al. applied the XFEM to investigate crack propagation patterns on log cross-sections, uncovering the relationship between energy release modes and crack length as well as quantity [8]. Additionally, experimental observation techniques have significantly advanced the understanding of drying-induced cracking in wooden components. For example, Botter-Kuisch et al. developed an experimental setup capable of monitoring the drying process in real time and correlating it with crack occurrence, providing data to reveal the critical conditions for cracking [9]. Hiroyuki et al. improved the CLSM system and conducted in situ observations of microcrack behavior during wood drying. They found that microcracks form when the surface moisture content falls below the fiber saturation point [10]. Antti used a laser reflection method to confirm that microcracks appear on the wood surface during the early stages of drying, and that higher temperatures and lower humidity levels lead to more severe microcrack propagation [11].

When drying cracks appear in timber beams serving as primary load-bearing structural members, the resulting degradation in load-bearing capacity becomes a central concern in engineering assessments. Among various evaluation methods, load-bearing capacity tests constitute the fundamental approach for obtaining direct insights. For instance, Bai et al. investigated the influence of crack depth and location on the side surfaces of timber beams on their bending performance [12]. Zhang et al. studied ancient timber beams with natural shrinkage cracks and developed degradation models for both load-bearing capacity and stiffness [13]. Anatoly examined the load-bearing capacity and stiffness of timber beams with through-thickness cracks, revealing that their performance depends not only on crack length but also on the position of the crack along the cross-sectional height [14]. Ivaniuk et al. investigated glued laminated timber beams with through-thickness cracks along the neutral axis, finding significant stress concentrations at crack tips and a decrease in load-bearing capacity with increasing crack length [15]. Using acoustic emission and digital image correlation techniques, Tu et al. studied the initiation and propagation of LT-type cracks in Chinese fir under three-point bending and found that the crack initiation load was approximately 40–50% of the peak load [16]. These studies collectively indicate that cracks of significant depth can severely reduce the bending strength and stiffness of timber beams and may alter their failure modes.

Existing studies have predominantly focused on isolated cracks or on assessing and repairing the overall performance of beams with existing cracks [17]. However, in naturally dried solid wood beams, cracks often do not appear singly due to stress release and redistribution during the drying process and exhibit certain spatial distribution patterns [18]. For example, the emergence of a deeper crack may locally release stress, thereby inhibiting the formation of new deep cracks in adjacent areas. When cracks appear simultaneously on both sides of a beam due to symmetrical drying, their combined effect may not be a simple superposition of the influences of two independent cracks [19]. Currently, there is a lack of systematic research on the mechanical interaction effects among such crack clusters with specific distribution patterns, as well as the comprehensive impact of these distribution characteristics on the ultimate load-bearing capacity of timber beams. Observations of historical timber structures [20] and modern drying experiments both suggest the existence of such distribution patterns; however, there is a shortage of integrated research that connects the initial drying stress field to the final assessment of load-bearing capacity.

The present study aims to systematically investigate the influence mechanisms and patterns of surface shrinkage cracks with typical distribution characteristics on the load-bearing capacity of timber beams. Systematic four-point bending tests are designed and conducted for several common surface crack distribution scenarios in engineering. By comparatively analyzing the ultimate load-bearing capacity and failure modes of timber beams under different crack distribution patterns, this study seeks to clarify the coupled effects of crack spatial distribution characteristics and depth on the bending performance of timber beams, particularly to verify and quantify the nonlinear relationship between the influences of double cracks and single cracks. This study aims to establish a correlation framework linking drying-induced damage characteristics to residual load-bearing performance, providing a more precise theoretical basis and data support for the scientific assessment, rational maintenance, and safe utilization of timber beams with shrinkage cracks, especially for historical timber components [21,22].

## 2. Materials and Methods

### 2.1. Material Selection

*Pinus sylvestris* var. *mongolica* was obtained from the Krasnoyarsk Krai region in Russia and used as the experimental material in this study. The material was characterized by a standard air-dry density of 480 kg/m^3^ [23]. This type of wood has the advantages of wide application, moderate strength, good durability, easy processing, and low cost; it is often used as a material for timber beams in timber structures [24,25]. To reduce the uncertainty in experimental results caused by individual differences in specimens, a large sample size was used in the experiments for each defect parameter, with a total sample size of 1200. In order to avoid excessive wood consumption and reduce the specimen processing difficulty and energy consumption, small-sized specimens were used for testing in this study.

### 2.2. Prediction of Shrinkage Crack Patterns

According to the principle of material mechanics, timber beams with a height greater than their width can better utilize their load-bearing capacity. A height-to-width ratio (*H*/*B*) of 1.5 is considered reasonable and is very common in beams of various timber structures. This study therefore begins with investigating the formation process and crack patterns of drying-induced shrinkage cracks in timber beams with this particular height-to-width ratio. To identify the crack configurations for subsequent load-bearing capacity experiments, a theoretical analysis was conducted to predict the possible crack patterns on timber beam cross-sections related to *R* direction cracks (The research sets the width of the timber beam along the R direction and the height along the T direction, which is the most common direction in historical building timber beams).

Considering a system of *n* interacting cracks propagate in *R*-direction of the wood beam cross-section with spacing *s* = *H*/*n* and at lengths *a*_1_, *a*_2_, *a_N_*_,_ with fracture energy Γ, the Helmholtz free energy has the following general form:

(1) $$H=U(a_{1},a_{2},\ldots ,a_{N})+\sum_{i=1}^{N}\Gamma da_{i}$$

where *U* is the elastic strain energy. There are many possible fracture equilibrium solutions, while the requirements for a crack system to evolve is that *H* should be minimized. The task’s goal is to determine the stable or unstable solution and then to obtain the stable solution [26].

The equilibrium and stability of crack system is decided by the first and second variations:

(2) $$\delta H=\sum_{i=1}^{m}\left(\frac{\partial U}{\partial a_{i}}+\Gamma \right)\delta a_{i}+\sum_{j=m+1}^{n}\left(\frac{\partial U}{\partial a_{j}}\right)\delta a_{j}$$

(3) $$\delta^{2}H=\frac{1}{2}\sum_{i=1}^{n}\sum_{j=1}^{n}\left(\frac{\partial^{2}U}{\partial a_{i}\partial a_{j}}\right)\delta a_{i}\delta a_{j}=\frac{1}{2}\sum_{i=1}^{n}\sum_{j=1}^{n}H_{jj}\delta a_{i}\delta a_{j}$$

where *i* = 1,…, *m* are the cracks that are propagating (*δa_i_* > 0), dissipating fracture energy Γ; *i* = *m* + 1,…, *n* are the cracks that are shortening (*δa_i_* > 0), for which the fracture energy is 0, and *i* = *n* + 1,…, *N* are the cracks that are arrested (*δa_i_* = 0), which occurs when the energy release rate −∂*U*/∂*δa_i_* is non-zero but less than the critical value.

Equilibrium crack propagation requires vanishing of the first parenthesized expression in Equation (2), which represents the Griffith crack propagation criterion of linear elastic fracture mechanics. There exist many equilibrium solutions that are reachable along a stable equilibrium path. Fracture stability requires the matrix of *H*_,*ij*_ equal to *U*_,*ij*_, to be positive definite:

Det *U*_,*ij*_ > 0 and *U*_11_ > 0 (4)

For the vectors of admissible variations *δa_i_*, the admissible crack length variations *δa*_i_ are those satisfying the following restrictions:

for ∂*U*/∂*a*_i_ = Γ: *δa_i_* ≥ 0 (5)

for 0 < ∂*U*/∂*a_i_* < Γ: *δa_i_* = 0 (6)

for ∂*U*/∂*a_i_* = 0: *δa*_i_ ≤ 0 (7)

In the special case of a parallel system of preexisting shrinkage cracks that open up to length *a_j_* but are closed beyond, the effective fracture energy is 0 and then:

for ∂*U*/∂*a_j_* = 0: any *δa_j_* (8)

**Figure 1:**
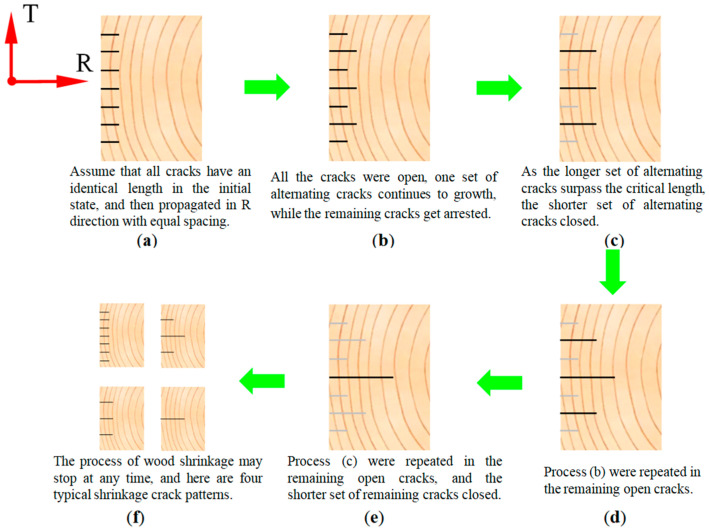
Schematic diagram of crack propagation process (taking the cracks formed on one side surface of a timber beam as an example). In the figure, dark black lines indicate the propagated or opened cracks; light gray lines indicate the closed cracks. The subfigures (**a**–**f**) in the figure represent the various stages of crack propagation from the initial formation of the crack to its final length.

Based on the above analysis, a schematic diagram of the crack propagation process on the cross-section of the timber beam can be drawn, as shown in Figure 1.

From the figure, it can be seen that, assuming that there are many parallel small cracks along the R direction on the left side of the cross-section of the timber beam at the initial stage, these cracks will continuously change during the drying and shrinkage process of the timber beam, as shown in Figure 1a–e. Some cracks will propagate, some cracks will not propagate, and some propagated cracks will even close again. Finally, four possible crack patterns evolved as shown in Figure 1f. In the T direction, theoretical Formulas (1)–(8) can also be used for prediction; four crack patterns similar to those in the R direction can also be obtained.

Although the above theoretical formula can predict the basic crack patterns that may occur on the cross-section of timber beams, it cannot be analyzed based on more specific parameter conditions (such as the moisture contents and drying processes that cause the cracks to occur). To make predictions based on environmental conditions, more theoretical knowledge and formulas are needed. Considering that predicting solely based on theory would require overly complex theoretical knowledge and formulas, the following finite element (FE) method was used for further prediction.

In the simulation, a timber beam with three-dimensional orthotropic material parameters was modeled. By defining initial moisture content and surface moisture evaporation flux, the crack evolution process under natural drying conditions was simulated. The relevant parameters for the drying shrinkage crack simulation are listed in Table 1.

The FE model simulated the drying shrinkage crack formation process in a timber beam with a cross-sectional dimension of 200 mm × 300 mm. Such beam dimensions were commonly used in historic timber structures; beams of this size are sufficient to develop a significant moisture gradient between the interior and the surface, making them prone to shrinkage cracking.

In this study, the purpose of the simulation is only to predict the most likely crack patterns to form on the cross-section of timber beams, and to analyze which parameters determine whether the timber beam will crack during the drying process. Therefore, it is not necessary to predict the moisture content in the timber beam very accurately at each time period. Therefore, it is more important to simplify the parameter settings in the simulation and make it easier for non-wood drying researchers to replicate the simulation process than to determine the precise values of each parameter.

To simplify the simulation, the temperature was assumed constant at 20 °C; under this condition the moisture diffusion coefficient (*MD*) of wood remains constant during drying. Considering that wood will only shrink continuously during the process of decreasing moisture content when its moisture content is lower than the fiber saturation point, the initial moisture content of the timber beam was set as the fiber saturation point, which is 30%. The boundary conditions of timber beams during the drying process were also set in the simplest way: when setting the boundary conditions, the moisture content of the atmosphere in the environment where the timber beams were located was not considered, but directly set according to the expected moisture content of the timber beam surface after the drying process was completed, which was 12%.

Another important parameter that can determine whether a timber beam will crack during the drying process is the evaporation flux (*Φ_v_*) on the surface of the beam, which is usually a function of air temperature, humidity, and air flow velocity. This parameter was also greatly simplified: five different orders of magnitude of *Φ_v_* are directly given (*Φ_v_* = {0.1, 1.0, 10, 100, 1000} × 10^−6^ kg·m^−2^s^−1^), and parameterized simulations were used to compare the effects on the drying and cracking process of timber beams when the value of *Φ_v_* decreased or increased.

In order to make the contour lines of the simulated humidity and stress fields smooth during the FE simulation process, fine grids were used in the simulation. A two-dimensional humidity–stress coupled element with four nodes was used for the simulation; all grids were set as standard squares with a side length of 2 mm. At this size, there are a total of 15,000 elements in the entire cross-section.

During the drying process, cracks may occur in the cross-section of timber beams. Although extended finite element method (XFEM) can be used to simulate the formation process of cracks; however, the coupling of multiple simulation methods can make the finite element model too complex. Therefore, in this study, only the coupling analysis of the humidity field and stress field is considered. When analyzing the influence of cracks on the stress field of the cross-section of a timber beam, a manual modeling method to establish a notch on the cross-section of the timber beam to simulate the crack was used. When using this modeling method, the FE program cannot automatically form cracks based on the simulation results of the stress field; however, it is still sufficient to simulate how the already formed cracks will cause changes in the stress field.

**Figure 2:**
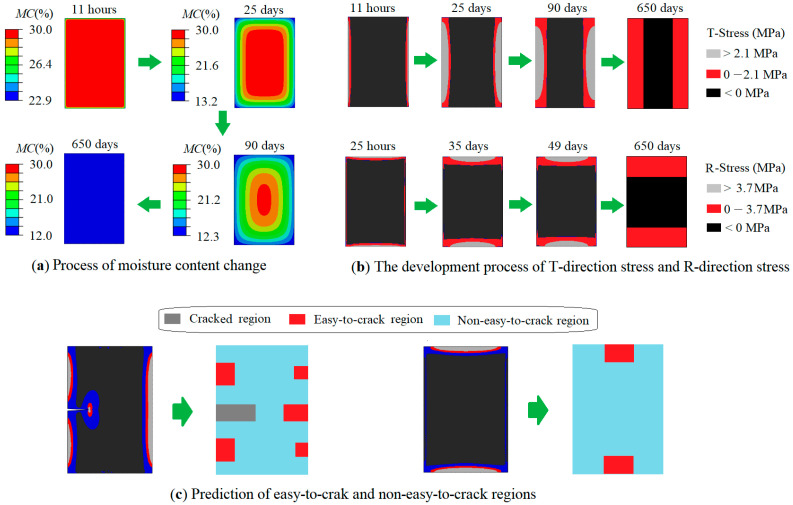
Schematic diagram of the prediction of crack patterns using the FE technique.

Figure 2a shows the variation process of moisture content on the cross-section of a timber beam under the condition of *Φ_v_* = 10 × 10^−6^ kg·m^−2^s^−1^.

As can be seen from the figure, each cross-sectional contour plot is marked with the corresponding drying time. When the drying time reaches 11 h, cracks begin to appear. At this time, the moisture content of the timber beam only decreases at the surface, and the depth of cracks are very small; when the drying time reaches 25 days, the stress reaches its maximum, and not only does the moisture content on the surface of the timber beam decrease, but its internal moisture content also decreases significantly. When the drying time reaches 90 days, the depth of the tensile stress region reaches its maximum (indicating that the crack depth is also likely to reach its maximum and it should be noted that in actual timber beams, the drying shrinkage process is likely to occur repeatedly, so cracks will be deeper than this prediction) and the overall moisture content of the timber beam further decreases at this time. When the drying time reaches 650 days, the drying process of the timber beam ends (the moisture content gradient in the entire wood is uniform, and the difference between the moisture content at the center and the surface is no more than 1.2%). The stress on the cross-section of the timber beam is also very low, and the cracks will not continue to propagate. When *Φ_v_* takes different values, the time required for each stage is shown in Table 2.

From the table, it can be seen that the water evaporation flux *Φ_v_* on the surface of the timber beam is a key parameter affecting the drying and cracking process of the timber beam; it determines whether the timber beam will crack and the time required for each drying stage, as follows.

When *Φ_v_* is small (e.g., *Φ_v_* = 0.1 × 10^−6^ kg·m^−2^s^−1^), the timber beam will not crack no matter how long the time. This is because the surface moisture evaporates slowly and the moisture gradient inside the timber beam is always small, resulting in uniform shrinkage inside and outside the beam; therefore, it will not crack.

Increasing *Φ_v_* (in practical situations such as increasing wind speed, reducing air humidity, etc.) leads to the cracking of timber beams during the drying process; the higher the value of *Φ_v_*, the shorter the time required for each stage. It can be seen that the change in *Φ_v_* has a more significant impact on the early stage of drying than on the late stage of drying. For example, when *Φ_v_* increases by an order of magnitude, the time of the first stage also decreases by an order of magnitude. However, the time reduction in stages 2, 3, and 4 is not that significant, especially when *Φ_v_* is already large (such as from 100 × 10^−6^ kg·m^−2^s^−1^ to 1000 × 10^−6^ kg·m^−2^s^−1^). Although *Φ_v_* increases by an order of magnitude, the times of stages 2, 3, and 4 only decrease by less than 10%; notably, the time of stage 4 only decreases by 1%. This is because the diffusion rate of water in wood is limited. When the surface evaporation rate is very high, even if the surface water evaporation flux continues to increase, it is difficult to accelerate the diffusion rate of internal water. Therefore, the total time required for drying will not be significantly reduced. The changes in the first stage mainly occur on the surface of the timber beam; therefore, an increase in *Φ_v_* will significantly reduce the time required for the first stage.

It should be noted that the simulated drying time mentioned above is only the time simulated under artificially set parameters and can be used to compare the relative length of time required for different drying stages. The physical quantities involved in the actual drying process are very complex and have not been carefully considered in the simulation model. Therefore, the simulated drying time can only be referenced in terms of magnitude; it is not equal to the time required for the actual drying process.

Figure 2b illustrates the distribution of drying stress across the beam cross-section under the assumption that no crack occurred. This assumption is necessary because once cracking initiates, the associated release of strain energy significantly reduces stress near the crack and alters the overall stress field within the timber beam.

Three distinct colors represent different stress levels in the figure: grey areas indicate high-tensile stress regions, and the stress is greater than the tensile strength of the wood in the corresponding direction; thus, cracks will initiate in this region. Red areas correspond to low-tensile stress regions, and the stress is less than the tensile strength of the wood in the corresponding direction. Therefore, it is difficult to generate new cracks in this region, but existing cracks may continue to propagate. Black areas correspond to compressive stress region, where neither crack initiation nor crack propagation takes place.

Figure 2c is a prediction of possible crack patterns that may form on timber beam cross-sections. The image on the left assumes that there is already a crack in the timber beam cross-section; it reveals that the stress behind the crack tip is released, resulting in a low tensile-stress region in the wake of the crack. Additionally, the stress in the regions adjacent to the crack flanks is reduced. Consequently, once a crack develops, it becomes unlikely for another crack of similar depth to form next to it. This observation is also consistent with the theoretical analysis results obtained through the strain energy formula earlier.

Observations of drying cracks in historic timber beams reveal that cracks can occur not only on one side face but also on the opposite side face, and sometimes on the top or bottom surfaces. The simulation results indicate that when cracks exist on two opposite faces of a beam, the stress magnitude at the crack tips is lower than that in beams with a crack on only one side. This suggests that when cracks are present on opposite faces, their depth tends to be shallower than in cases with a single-side crack.

Through simulation, the most probable locations for crack initiation in a timber beam were predicted. These critical positions typically include the mid-height of the side faces, the quarter-height and three-quarter-height points on the sides, and the center of the top and bottom surfaces. Based on the prediction of crack locations mentioned above, specimens for testing the load-bearing capacity of timber beams were designed.

### 2.3. Experimental Methods and Measurement Data Correction Methods

Based on the aforementioned predictions regarding the most probable locations of drying cracks in a timber beam cross-section, small, defect-free specimens were artificially pre-cracked; four-point bending fracture tests were conducted to evaluate the changes in load-bearing capacity of timber beams after drying-induced cracking. While full-scale bending fracture tests on dried and cracked beams could more directly illustrate the influence of drying cracks on load-bearing capacity, such an approach is impractical for large sample size statistical analysis due to the high costs and substantial material requirements. Therefore, this study employed small-sized specimens to achieve a large sample size for experimental investigation.

**Figure 3:**
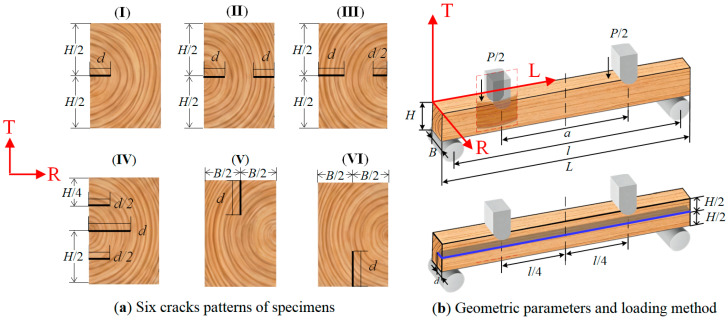
Crack patterns, geometric parameters, and loading method of specimens.

Figure 3a illustrates the six different crack patterns of specimens adopted in the tests. For each crack pattern, specimens were prepared with pre-cracks of varying depths to enable comparative analysis. Figure 3b provides an explanation of the geometric parameters and loading method of the specimens.

In the experiments, the crack depth settings for six crack pattern specimens in Figure 3a are shown in Table 3. The design of the pre-crack depth is based on two considerations: to first facilitate the processing of prefabricated crack specimens; and that there should be typicality to facilitate parameterized comparisons in experiments. It should be noted that in real, dry, cracked timber beams, cracks may not necessarily reach the same depth. The purpose of the experiment is to clarify the effects of cracks at different depths, so as to facilitate engineers to further design full-scale timber beam experiments.

In order to facilitate the comparison of experimental results and minimize the uncertainty of experimental results caused by minor variations in specimen dimensions and densities, this study limits the relationship between the span and height of timber beams to *l* = 10 *H*, and uses specimens with uniform dimensions and spans (*l*_0_ = 150 mm, *H*_0_ =15 mm, *B*_0_ = 10 mm, *L*_0_ = 160 mm). In the experiments, a four-point bending loading method with a loading point distance of half the span of the timber beam was used to test the timber beam; the maximum load value before the fracture of each timber beam was measured.

In the preparation stage of the test piece, 80 specimens without macroscopic defects, as well as 1120 specimens with different longitudinal-crack parameters were processed. Specimens with longitudinal cracks were prepared using a special method: an ultra-thin small diameter saw blade with a thickness of only 0.5 mm was used to fabricate the cracks.

**Figure 4:**
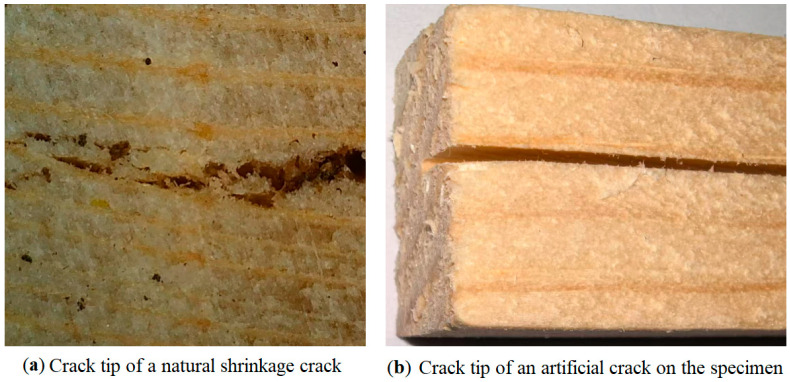
Comparison of microscopic images of crack tip between a natural shrinkage crack and an artificial crack.

It should be noted that the experimental conditions differ from the natural shrinkage cracks formed in timber beams during actual service. Natural shrinkage cracks typically exhibit more irregular tip morphologies, and the crack surfaces may present a “fiber bridging” effect due to partially unbroken wood fibers connecting across the crack faces (see Figure 4). Fiber bridging consumes additional fracture energy, which may delay crack propagation and thereby alter the rate of load-bearing capacity degradation.

However, it is impossible to produce a large number of specimens with identical crack sizes and positions by prefabricating cracks using natural drying methods in experiments with large sample sizes, which makes the reproducibility of the experiment very poor. The cracks prefabricated by manual sawing processing can maintain a high degree of consistency in the shape of the cracks in the sample, which is conducive to conducting parametric experimental research based on a large sample size.

In this study, based on the prediction of the possible location of natural shrinkage cracks, while taking into account the convenience of large-scale experimental specimen production and the repeatability of experiments, cracks were usually prefabricated at positions that satisfy geometric symmetry on the specimens. This cannot be exactly the same as naturally formed cracks, particularly because artificial cracks do not exhibit “fiber bridging”. It can be seen that the predicted results of this experimental method are more “dangerous” than those of natural cracks. This is not bad for engineers; the third strength theory of materials mechanics overestimates dangerousness. Therefore, the engineering designs based on the predicted results of this study will be safer.

The processed specimens were stored in a dedicated drying oven with a moisture content controlled at 12.0% ± 1.5%.

Before starting the experiment, a KT50 upgraded inductive wood moisture meter (Jingtai Instrument Co., Ltd., Xinghua, China) was used to retest the moisture content of the specimens (4 × 5 specimens are bundled and stacked for measurement to make the measurement results of moisture content more accurate) and ensure that the moisture content of specimens meets the requirements (12.0% ± 1.5%). Then, the cross-sectional width (*B*), cross-sectional height (*H*), and total length (*L*) of specimens were measured using a vernier caliper; the static mass (*m*) of the specimens were measured using an electronic balance with a resolution of 0.01 g. Taking 40 specimens in the experiment as an example, the variations in their size and weight parameters are shown in Table 4.

From the data in the table, it can be seen that the size variation among the specimens is very small, while the mass variation is slightly larger, but still much smaller than the variation in the mechanical properties of the wood. It can be seen that the size of the specimens is relatively stable. Although there was a greater difference in mass of the specimens, specimens with excessive or insufficient mass were excluded in the early stages.

During the experiment stage, specimens were placed on a dedicated metal hinge support with a span of *l* = 150 mm, and subjected to fracture and failure loading using a universal mechanical testing machine, PUYAN980 (Yaofeng Electronic Equipment Co., Ltd., Dongguan, China), with a maximum load of 20 kN and load measurement resolution of 0.01 N, in conjunction with a four-point bending loading head.

The testing machine used in the experiment meets the accuracy standards of ordinary mechanical testing machines; within the entire measurement range, its load measurement error does not exceed ±0.5%. Compared to the changes in load-bearing capacity caused by differences in the mechanical properties of wood, the error of the mechanical testing machine was negligible. Therefore, in this study, no additional analysis was conducted on the errors caused by the mechanical testing machine.

First, the specimens without macroscopic defects were tested. Subsequently, the specimens with pre-cracks were tested and the maximum loading-force (*P*) of each specimen during the fracture process was recorded. To reduce the uncertainty of the experimental results caused by differences in the size and density of specimens, it is necessary to adjust the tested loading-force *P* to an equivalent value *P*_e_:

*P*_e_ = *P*·C*_ρ_*·C_W_ (9)

where C*_ρ_* is the density influence coefficient used to adjust the impact of density differences on experimental results; and *C*_W_ is the coefficient of influence on cross-sectional dimensions used to adjust the impact of machining deviations on experimental results. The physical meaning of the equivalent fracture load value is the fracture load value of a specimen with standard density and identical cross-sectional dimensions, which is calculated based on the assumption that the load-bearing capacity of the specimen is proportional to its density.

The calculation formula for the density influence coefficient *C_ρ_* is:

*C_ρ_* = *ρ*_0_/*ρ* (10)

where *ρ*_0_ is the standard air-dry density of *Pinus sylvestris* var. *mongolica* wood (taken as 480 kg/m^3^); and *ρ* is the actual air-dry density of the specimen. For defect-free specimens and those with pre-cracks, the specific calculation formula for *ρ* can be calculated separately through Formulas (11) and (12):

*ρ*_(defect-free-beam)_ = *m*/*BHL* (11)

*ρ*_(cracked-beam)_ = *m*/(*BHL* − ∑*dδL*) (12)

where *B*, *H*, *L* and *m* are the width, height, length and the static mass of specimens, respectively, and *δ* is the width of the prefabricated crack (the volume of the prefabricated crack is *δdL* and in calculating, take *δ* = 0.5 mm). It can be seen that the calculation formulas take into account the volume of wood removed due to the fabrication of cracks, making the calculated density more accurate. In addition, when measuring the static mass of specimens, based on the condition of a moisture content of 12.0% ± 1.5%, it is ensured that the calculated *ρ* is the actual air-dry density of the specimens.

The calculation method for the influence coefficient *C*_W_ of the cross-sectional dimensions is shown in Formula (13):

*C*_W_ = *B*_0_*H*_0_^2^/(*BH*^2^) (13)

where *B*_0_ and *H*_0_ are the standard width and height of the specimen (*B*_0_ = 10.0 mm, *H*_0_ = 15.0 mm), respectively; and *B* and *H* are the actual cross-sectional width and height of the specimen measured by a vernier caliper (accuracy 0.1 mm).

By introducing the density influence coefficient and cross-sectional influence coefficient to adjust the experimental results, the influence of density differences between specimens and processing errors in specimen size on the experimental results can be significantly reduced. Examples of the fracture load adjustment in this study are shown in Table 5. Case No.1 is a defect-free specimen; therefore, the total depth of its cracks is ∑*d* = 0.0 mm. Case No.2 is a specimen with a single crack and the crack depth is 3.0 mm; therefore, the total depth of the cracks is ∑*d* = 3.0 mm. Case No.3 is a specimen with three cracks, with crack depths of 6.0 mm, 3.0 mm, and 3.0 mm, respectively. Therefore, the total depth of the cracks is ∑*d* = 6.0 mm + 3.0 mm + 3.0 mm = 12.0 mm.

From the process of adjusting the fracture load in the table, it can be seen that if the cross-sectional size and density of the timber beam are below the standard value, the adjusted fracture load value will increase compared to the actual measured value. If the cross-sectional size and density of the timber beam are exceeding the standard value, the adjusted fracture load value will decrease compared to the actual measured value. By using this method for adjustments, the influence of size and density differences between specimens on experimental results can be reduced, making the experimental results more credible.

Despite the use of adjustment methods, individual differences in the mechanical properties of timber beams still induce uncertainty in experimental results. This study aimed to reduce the influence of random factors by using a large sample size.

Based on prior research and the empirical evidence from this study, four typical conditions of data dispersion were identified. In Table 6, for each condition, the coefficient of variation (*CV*) is provided and the necessary sample size to achieve a confidence interval width of either ±5%, ±7.5% or ±10% at the 95% confidence level is given.

In this study, the target width of the confidence interval was defined relative to the experimental value. From Table 6, it can be seen that when the target width is ±7.5%, a sample size of 40 can meet the experimental requirements for all cases, ensuring both a reasonable sample size and experimental accuracy. Therefore, in this study, a sample size of 40 was used for all specimen groups.

Considering the large amount of data in this study, the research team used a self-developed script combined with Excel 2019 to input, organize, calculate, and analyze key experimental data such as specimen size (*B*, *H*, *L*), mass (*m*), density (*ρ*), density influence coefficient (*C_ρ_*), cross-sectional size influence coefficient (*C*_W_), fracture load (*P*) and its equivalent value (*P*_e_) (including maximum, minimum, average, and coefficient of variation). Origin2019b software was utilized to perform the statistical analysis, as well as to generate the curve plots and box plots.

### 2.4. Quantitative Description Method for Load-Bearing Capacity of Defective Timber Beams

In order to facilitate load-bearing capacity evaluation in experiments, three coefficients were defined in this study: the “load-bearing capacity coefficient (*R*)”;“decline ratio of load-bearing capacity (*D*)”; and “comprehensive crack-influence coefficient (*β*)”.

The physical meaning of the load-bearing capacity coefficient is the ratio of the equivalent fracture load value of defective timber beams to the fracture load value of defect-free timber beams under the same conditions. Its calculation formula is:

*R* = *P*_ed_/*P*_e_ (14)

where *P*_ed_ is the equivalent fracture load value of a defective timber beam and *P*_e_ is the equivalent fracture load value of a defect-free timber beam. Due to the fact that the fracture load value of defective timber beams is usually smaller than that of defect-free timber beams, *R* is usually less than 1.0.

The physical meaning of the decline ratio of load-bearing capacity is the rate of reduction in the load-bearing capacity of the timber beam caused by cracks. Its calculation formula is:

*D* = (1 − *R*) × 100% (15)

The comprehensive crack-influence coefficient is defined in Formula (16):

*β* = *DB*/(Σ*d_i_*, *i* = 1, 2,…, *n*) (16)

where *D* is the decline ratio of load-bearing capacity and Σ*d_i_* denotes the sum of the depths of all cracks present on the specimen (assuming a total of *n* cracks).

Physically, this coefficient represents the normalized decline ratio of the load-bearing capacity, which is scaled to the crack depth equivalent to the beam width, and enables the comparison of loading-bearing performance of specimens with different crack patterns.

## 3. Results

### 3.1. Load-Bearing Capacity of Crack-Pattern-I Specimens with Different Crack Depths

In this comparative experiment, 80 crack-free specimens were measured; the average fracture load was taken as the reference value. The average equivalent fracture load of 80 crack-free (*d*/*B* = 0) specimens was 1473 N, with a maximum value of 2159 N and a minimum value of 884 N. The *CV* value was 18.1%, higher than that of the pre-cracked control groups. The reason for the relatively high *CV* is that *Pinus sylvestris* var. *mongolica* wood is mainly composed of tubular cells at the microscopic level; there are natural differences in cell wall thickness, arrangement direction (fiber angle), and chemical composition (such as cellulose and lignin) among the samples. The random distribution of microstructures and defects directly leads to a significant dispersion of fracture loads, which is the fundamental reason for the relatively high *CV*.

The experimental data for crack depth ratios, ranging from 0.20 to 0.90, are presented in Table 7. Since the reduction in load-bearing capacity is not significant when the crack depth ratio is small, only cases with a crack depth ratio of *d*/*B* ≥ 0.20 were measured.

One-way ANOVA revealed that crack depth had an extremely significant effect on *R*-value (*F*(10, 429) = 35.5, *p* < 0.001). Results from the Tukey post hoc test reveal that the influence of crack depth exhibits distinct stages: the group with the smallest crack depth (*d*/*B* = 0.20) has the highest load-bearing capacity coefficient (Group A); as crack depth increases (*d*/*B* = 0.25, 0.30, 0.35, 0.40, 0.45, 0.50, 0.60, 0.70, 0.80), the load-bearing capacity coefficient decreases accordingly (Group B, C, D, E, F); the group with the deepest crack depth (*d*/*B* = 0.90) also has the lowest load-bearing capacity coefficient (Group G).

**Figure 5:**
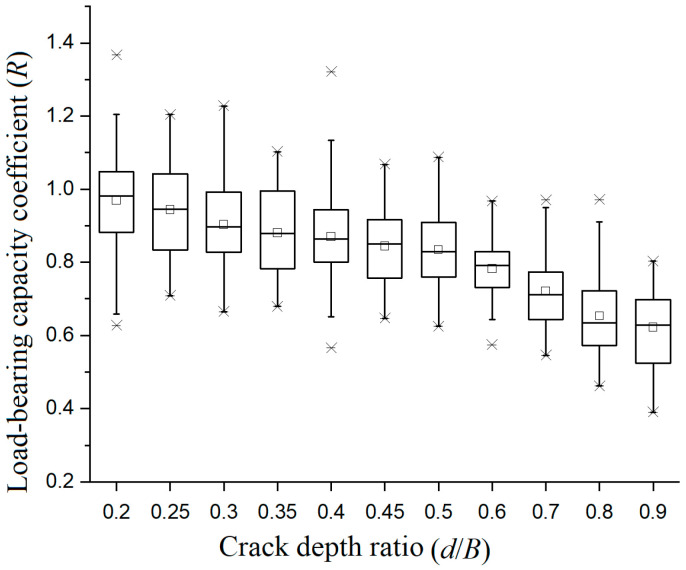
Box plot of the load-bearing capacity coefficient of crack-pattern-I specimens under different crack depth conditions.

Figure 5 is the box plot of crack pattern-I specimens, it can be observed that the load-bearing capacity coefficient of specimens decreases monotonically with increases in the crack depth ratio. When the crack depth ratio is low, the median is close to the defect-free specimen group; when the crack depth ratio is high, it drops to the range of 0.6 to 0.7. Under different crack depth ratios, the height of the box is relatively stable and the average and median values are also close, indicating that the degree of data dispersion is basically consistent.

The length of the upper and lower lines is basically balanced, with a small number of outliers and a random distribution. The overall pattern indicates that the attenuation trend is relatively uniform; there is no obvious critical point of sudden change. For actual timber beams, it is recommended to control the crack depth ratio at a lower level to maintain high load-bearing performance. When the crack depth ratio is high, it is necessary to strengthen monitoring and prevent the risk of deterioration of the load-bearing capacity.

**Figure 6:**
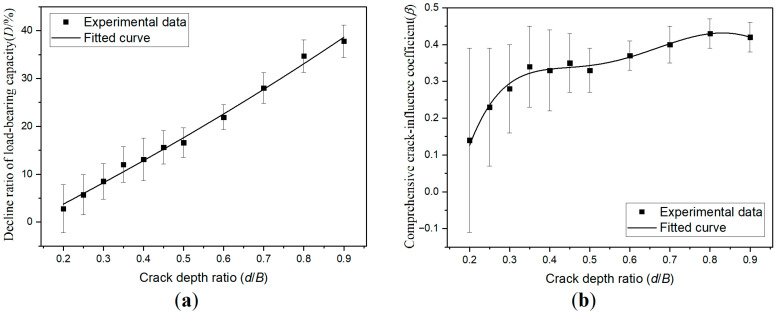
Load-bearing performance of crack-pattern-I specimens under different crack depth conditions: (**a**) decline ratio of load-bearing capacity; and (**b**) comprehensive crack-influence coefficient. The error bars in the figure are plotted with a 95% confidence interval. In the following curve graphs, meaning of error bars are also the same as in this figure.

Figure 6 shows the experimental data fitting curve of load-bearing performance. It can be observed form Figure 6a that when the crack depth is one-fifth of the specimen’s thickness, the decline ratio of load-bearing capacity is less than 5%. In engineering practice, it can be considered that cracks of this depth have little effect on the load-bearing capacity of timber beams. Once the crack depth approaches one-third of the specimen thickness, the decline ratio of load-bearing capacity increases to about 10%. It can be considered in engineering practice that such deep cracks have a significant impact on the load-bearing capacity of timber beams and require manual intervention.

Contrary to intuition, even the crack depth reaches 90% of the specimen thickness, the specimen does not lose most of its load-bearing capacity; the reduction remains under 40%. This is because the crack nearly divides the specimen into two separate mini-beams, each of which retains considerable load-bearing ability, enabling the entire specimen to maintain more than half of its original load-bearing capacity.

The curve shape in Figure 6a is close to a straight line, from which an approximate estimation formula for the relationship between the decline ratio of the load-bearing capacity and the depth of the crack can be obtained:

*D* ≈ 2.8% + (*d*/*B* − 0.2) × 50% (17)

It should be noted that, although the curve appears approximately linear, the effect can grow exponentially. For example, when the crack depth ratio is 0.3, the decline ratio of the load-bearing capacity is only 5.7%; however, when the crack depth ratio reaches 0.6, the decline ratio of the load-bearing capacity increases to 21.9%. This indicates that a twofold increase in crack depth leads to a far more than twofold decline in the ratio of the load-bearing capacity. The comprehensive crack-influence coefficient curve in Figure 6b reflects this exponential growth trend. However, when the crack depth ratio exceeds 0.6, the comprehensive crack-influence coefficient curve becomes smoother.

### 3.2. Load-Bearing Capacity of Crack-Pattern-II and Crack-Pattern-III Specimens with Different Crack Depths

Table 8 presents the experimental data for crack-pattern-II and -III specimens. When crack-1 and crack-2 have an identical depth, the specimen is classified as a crack-pattern-II specimen; when crack-1 and crack-2 have different depths, the specimen is classified as a crack-pattern-III specimen.

**Figure 7:**
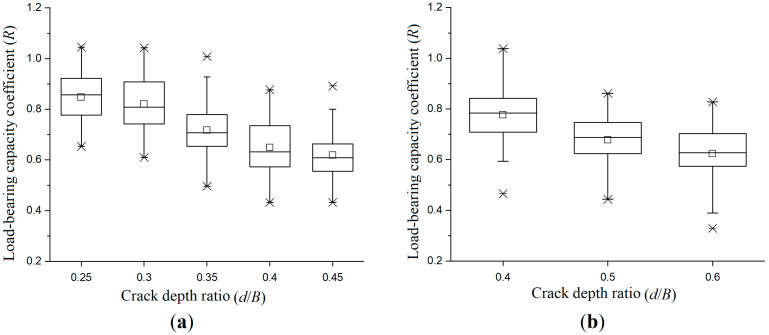
Box plot of load-bearing capacity coefficient under different crack depth conditions: (**a**) crack-pattern-II specimens; and (**b**) crack-pattern-III specimens.

Figure 7 is the load-bearing capacity box plot of crack pattern-II and -III specimens. It can be observed that the dispersion of the experimental data is relatively consistent. The height of the box with crack-pattern-III specimens increases with the increase in the crack depth ratio. In the dataset with a low crack depth ratio, the dispersion degree significantly increases with higher crack depth ratios; the lower whisker line is significantly extended. The comparison of the two sub-figures shows that the data uncertainty of crack-pattern-III specimens is higher at high crack depth ratios. In engineering, stricter safety margins and monitoring mechanisms need to be established for timbers with this crack pattern.

**Figure 8:**
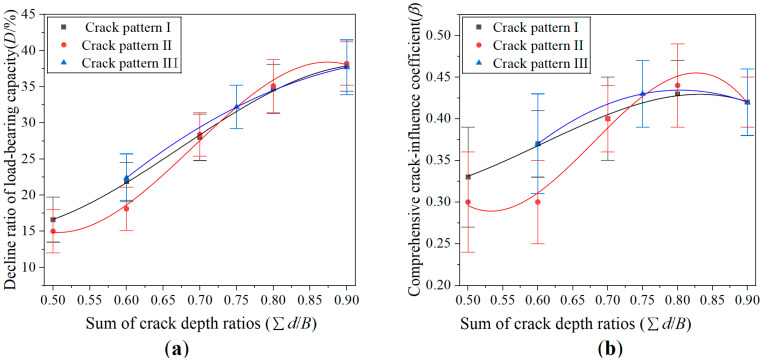
Load-bearing performance of crack-pattern-I, -II, and -III specimens under different crack depth ratios: (**a**) decline ratio of load-bearing capacity; and (**b**) comprehensive crack-influence coefficient.

Figure 8 compares the crack pattern-I, -II, and -III specimens, where Figure 8a,b shows the experimental data fitting curves of the decline ratio of load-bearing capacity and comprehensive crack-influence coefficient, respectively. As shown in Figure 8a, under the same total crack length, the decline ratios of load-bearing capacity for crack-pattern-I and -III specimens are close, but the decline ratio of crack-pattern-II specimens is slightly lower. This is attributed to the fact that crack-pattern-II specimens exhibit the best symmetry in the positions of the two cracks, resulting in the lowest decline ratio of load-bearing capacity among the three patterns. From Figure 8b, it can be observed that under identical total crack lengths, the comprehensive crack-influence coefficient of crack-pattern-II specimens is also the lowest, while those of crack-pattern I and III specimens are nearly identical. Both sets of curves indicate that the overall differences among crack-pattern-I, -II, and -III specimens are relatively small. This is primarily due to the same vertical positions of the cracks and the identical orientation of the cracks in specimens with these three crack patterns.

### 3.3. Load-Bearing Capacity of Crack-Pattern-IV Specimens with Different Crack Depths

Table 9 presents the experimental data for crack-pattern-IV specimens. There are three pre-cracks on each specimen: the crack at middle-height position of the specimen is the primary crack (crack-1 in the table), which is the deepest. The other two cracks located at 1/4 and 3/4 height of the specimen, respectively, are secondary cracks (crack-2 and crack-3 in the table). The depth of the secondary cracks is only half that of the primary crack.

**Figure 9:**
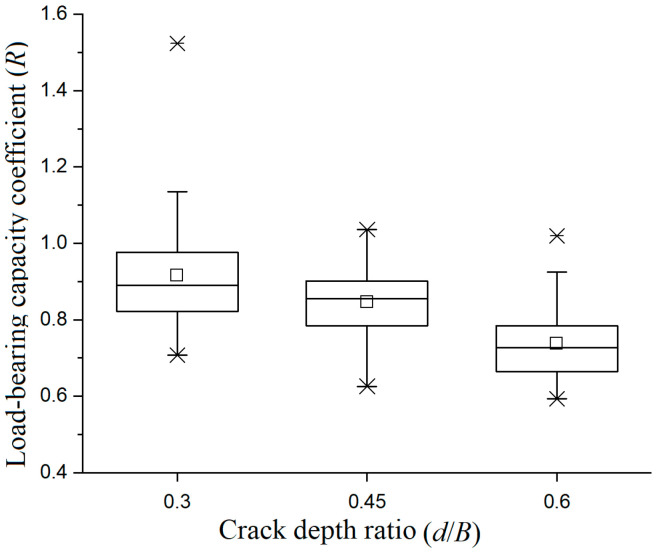
Load-bearing capacity coefficient box plot of crack-pattern-IV specimens under different crack depth conditions.

Figure 9 is the load-bearing capacity box plot of crack pattern-IV specimens under three different crackdepth conditions. From the box plot, it can be seen that the overall stability of the three sets of data is good. The second and third sets have flat boxes, short whiskers, and low dispersion. The first group has slightly higher relative dispersion, but still within a reasonable range, and the data quality is reliable.

**Figure 10:**
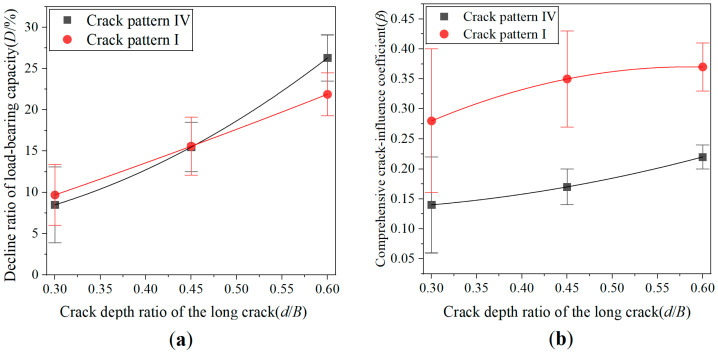
Load-bearing performance of crack-pattern-I, -IV specimens under different crack depth conditions: (**a**) decline ratio of load-bearing capacity; and (**b**) comprehensive crack-influence coefficient.

The variation in the decline ratio of the load-bearing capacity and comprehensive crack-influence coefficient with crack depth was plotted as experimental data fitting curves and compared with the curve of the crack-pattern-I specimens, as shown in Figure 10.

As can be seen from Figure 10a, when the depth of the deepest crack is less than 0.45 times of the specimen width, the decline ratio of the load-bearing capacity of crack-pattern-IV specimens is very close to that of crack-pattern-I specimens. This indicates that secondary cracks in crack-pattern-IV specimens do not significantly weaken the load-bearing capacity. When the depth of the deepest crack exceeds 0.45 times of the specimen width, the decline ratio of the load-bearing capacity for crack-pattern-IV specimens becomes higher than that of crack-pattern-I specimens. This suggests that when the crack is relatively deep, secondary cracks in crack-pattern-IV specimens contribute to a reduction in the load-bearing capacity.

As can be observed from Figure 10b, regardless of the crack depth, the comprehensive crack-influence coefficient of crack-pattern-IV specimens is considerably lower than that of crack-pattern-I specimens. This is because the total crack depth in crack-pattern-IV specimens are twice of that in crack-pattern-I specimens, while the corresponding increase in the decline ratio of the load-bearing capacity is relatively limited. Therefore, the influence of unit crack depth is significantly reduced.

### 3.4. Load-Bearing Capacity of Crack-Pattern-V and Crack-Pattern-VI Specimens with Different Crack Depths

Table 10 presents the experimental data for crack-pattern-V and -VI specimens. It should be noted that the cracks of these two specimens are located on the upper and lower surfaces of the specimens, respectively. However, in order to facilitate the comparison of crack depth with specimens of other crack patterns, the ratio of crack depth to specimen height (*d*/*H*) was not used to describe the crack depth. The ratio of crack depth to specimen width (*d*/*B*) is used in the table.

**Figure 11:**
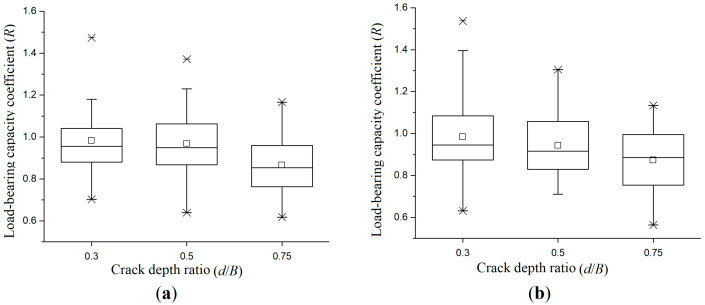
Box plot of load-bearing capacity coefficient under different crack depth conditions: (**a**) crack-pattern-V specimens; and(**b**) crack-pattern-VI specimens.

Figure 11 is the load-bearing capacity box plot of crack pattern-V and -VI specimens. Compared with crack-pattern-I to -IV specimens, the data dispersion of crack-pattern-V and -VI specimens are greater. This is due to the fact that the cracks are located on the top and bottom surfaces of specimens, indicating that the cracks at these positions will increase the uncertainty of the experimental results to a certain extent; When the crack is located at the side surface, the data are more stable. However, the data variation range of crack-patterns-V and -VI specimens are still within the normal range, and the data are valid and available.

**Figure 12:**
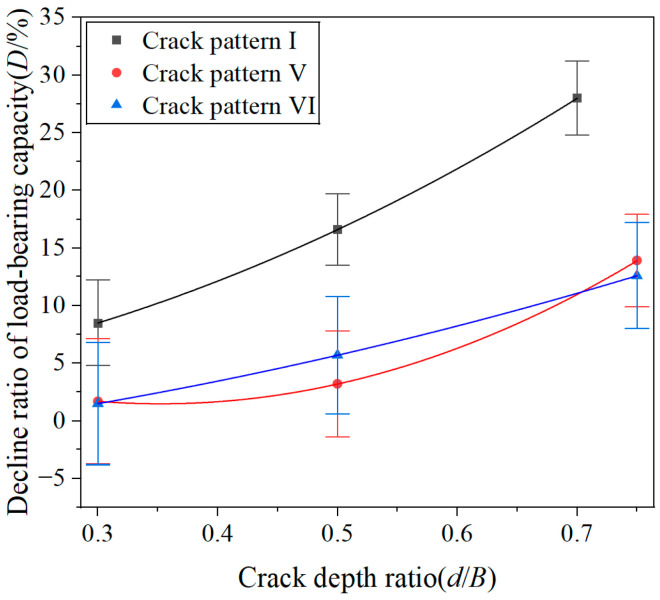
Relationship between the decline ratio of load-bearing capacities and the crack depth ratios for crack-pattern-I, -V, and -VI specimens.

The decline ratio of the load-bearing capacity curves and comprehensive crack-influence coefficient curves of crack pattern-V and -VI specimens are plotted in Figure 12, as well as a comparison with crack-pattern-I specimens. It can be observed from the figure that, regardless of the crack depth, the comprehensive crack-influence coefficients for crack-pattern-V and -VI specimens are significantly lower than those for crack-pattern-I specimens. This indicates that, for the same crack depth, cracks located at the top and bottom surface of specimens have weaker influence on the load-bearing capacity as compared to cracks located on the side surfaces of specimens.

## 4. Discussion

### 4.1. The Influence Mechanism of Cracks on Load-Bearing Capacity

**Figure 13:**
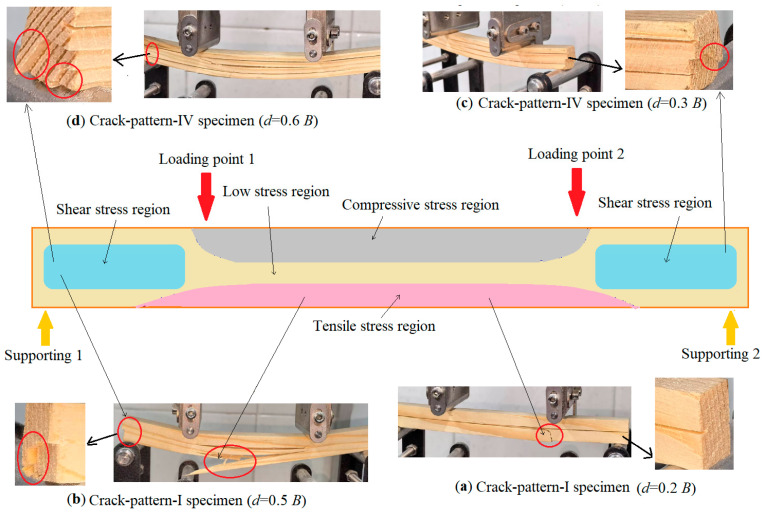
Stress contour plot in timber beams under the loading condition of four-point bending and fracture morphology of specimens.

In this study, four-point bending tests were systematically designed and conducted to investigate the influence of different surface shrinkage crack patterns on the load-bearing capacity of timber beams. The experimental results established the relationships between the crack depth, location and distribution patterns, and the beams’ load-bearing performance, while also revealing some mechanisms through which shrinkage cracks affect load-bearing capacity. The fracture morphology photos and fracture mechanism analysis of crack-pattern-I and crack-pattern-IV specimens are shown in Figure 13.

A comparison of crack-pattern-I specimens with a 20% width crack and a 50% width crack is shown in Figure 13a,b. It can be observed, in both situations, that wood fibers below the crack were broken. These fibers are in the tensile stress region, indicating that wood fibers have been pulled apart. However, with the 20% width crack, no shear cracking occurred at the end of the specimen, while with the 50% width crack, shear cracking occurred at the end of the specimen.

It was observed that when the crack depth exceeds one-third of the specimens’ width, the probability of shear failure of specimens increases significantly. This phenomenon can be attributed to the reduction in the effective cross-sectional area due to increased crack depth, as well as a shift in the failure mode of the beam from tension-dominated fiber failure with shallow cracks to shear-dominated failure with deep cracks. It is worth mentioning that, even when the crack depth reaches 90% of the beam width, specimens still retain approximately 60% of its original load-bearing capacity. This observation differs from Ivaniuk’s study, which concluded that “deep cracks lead to the loss of most of the structural load-bearing capacity”.

In fact, under four-point bending, although the crack nearly separates the cross-section, the remaining parts can still function as two smaller beams working together to share the load. According to the principles of material mechanics, a crack at the mid-height layer transforms a beam with section height *H* into two superimposed beams, each with a section height of *H*/2, resulting in a total twofold decrease in the section flexural modulus *W*_z_, critically weakening its integrity and resulting in the lowest load-bearing capacity. However, according to calculations in material mechanics, this type of timber beam split in half by a crack can still maintain 50% of its load-bearing capacity.

A comparison of crack-pattern-IV (multiple cracks) specimens with a 30% width crack and a 60% width crack is shown in Figure 13c,d. The experimental results indicate that when the depth of the primary crack was small (30% of the specimen’s width), failure occurred primarily in the form of shear failure induced by the primary crack, and two secondary cracks (15% of the specimen width) did not propagate. Thus, the influence of two secondary cracks on the overall load-bearing capacity was almost negligible. However, when the primary crack depth was larger, both the primary crack (60% of the specimen width) and two secondary cracks (30% of the specimen width) propagated under shear stress.

This indicates that when there are multiple cracks with different depths on a timber beam, secondary cracks can sometimes weaken the load-bearing capacity. Based on the above analysis, it can be seen that, in actual engineering evaluations, engineering personnel cannot only focus on the deepest crack; on the contrary, they must consider the comprehensive impact of the overall crack distribution.

### 4.2. The Mechanical Mechanism of Different Effects of Symmetric and Asymmetric Cracks

**Figure 14:**
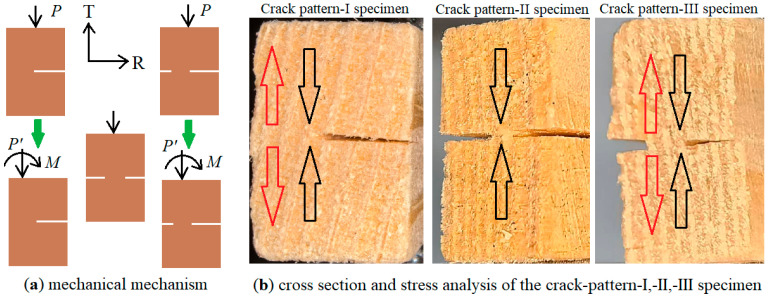
T-direction stress analysis from the perspective of combined deformation: in subfigure (**a**), *P* represents the original load received by the specimen, *P*′ represents the concentrated force after the decomposition of the original load, and *M* represents the bending moment after the decomposition of the original load; and in subfigure (**b**), the red hollow arrow represents tensile stress, and the black hollow arrow represents compressive stress.

Through the analysis of experimental data, it was found that under the same total crack depth, different crack distribution patterns may have varying effects on load-bearing capacity. The decline ratio in load-bearing capacity for pattern-II (symmetrical double cracks) specimens is slightly lower than that for pattern-I (single crack) and pattern-III (asymmetrical double cracks) specimens. The mechanical mechanism of this observation can be explained from the perspective of combined deformation of material mechanics, as shown in Figure 14.

From Figure 14a, it can be seen that the crack-pattern-I (single crack) specimen and the crack-pattern-III (asymmetric double cracks) specimen undergo bending deformation and compression in the TR plane, while the crack-pattern-II (symmetric double cracks) specimen only undergoes compression in the TR plane.

The situation of T-direction stress on the cross-sections of specimens is shown in Figure 14b. According to the principle of combined deformation in material mechanics, specimens that undergo bending deformation in the TR plane will have a much higher maximum tensile stress in the T-direction on their cross-sections than specimens that do not undergo bending deformation in the TR plane. The specific calculation process of T stress is as follows.

According to the translation theorem of force lines in theoretical mechanics, the calculation formulas for bending moment *M* in crack-pattern-I and -III specimens are represented by Formulas (18) and (19), respectively:

*M*_(crack-pattern-I)_ = *Pd*/2 (18)

*M*_(crack-pattern-III)_ = *Pd*/4 (19)

The maximum stress in the T direction generated by bending within the TR plane is:

*σ*_Tmax_ = *M*/*W*_L_ (20)

where *W*_L_ is the bending section coefficient of the specimen bending around the L axis in the TR plane. For the crack-pattern-I and -III specimens, *W*_L_ is represented by Formulas (21) and (22), respectively:

*W*_L(crack-pattern-I)_ = *L*(*B* − *d*)^2^/6 (21)

*W*_L(crack-pattern-III)_ = *L*(*B* − 1.5*d*)^2^/6 (22)

Unlike the crack-pattern-I and -III specimens, crack-pattern-II specimens do not bend in the TR plane and, therefore, only experience compressive stress with a magnitude of:

*σ*_T(crack-pattern-II)_ = *P*/*L*(*B* − 2*d*) (23)

Using the above formula, three cases with a total crack depth of 0.3*B*, 0.6*B*, and 0.9*B* were selected to calculate the maximum tensile and compressive stresses in the T direction in the crack-pattern-I, -II, and -III specimens, respectively. For the convenience of understanding the magnitude of stress, the maximum stress in the calculated specimen was converted into a multiple of the compressive stress in the crack-pattern-II specimen, as shown in Table 11.

From the data in the table, it can be seen that the crack-pattern-II specimen only experiences compressive stress in the T direction and no tensile stress. The other two specimens with asymmetric crack positions not only have compressive stress but also tensile stress in the T direction; when the total depth of the crack is large, both the tensile stress and compressive stress in the T direction in the specimens with asymmetric crack positions can reach many times that of the specimens with symmetric crack positions. According to wood science, the tensile strength of wood in the T direction is the lowest among all directions. Therefore, in asymmetric specimens, the additional tensile stress caused by the bending of the TR plane makes the asymmetric specimens more prone to failure.

### 4.3. The Influence of Crack Location

The experimental results clearly indicate that cracks on the side surfaces of timber beams have a far more significant influence on load-bearing capacity than those on the top or bottom surfaces. This is primarily due to the non-uniform distribution of bending stress along the cross-sectional height, which creates a stress gradient in the vertical direction, as well as the greater susceptibility of side-surface cracks to induce shear failure.

**Figure 15:**
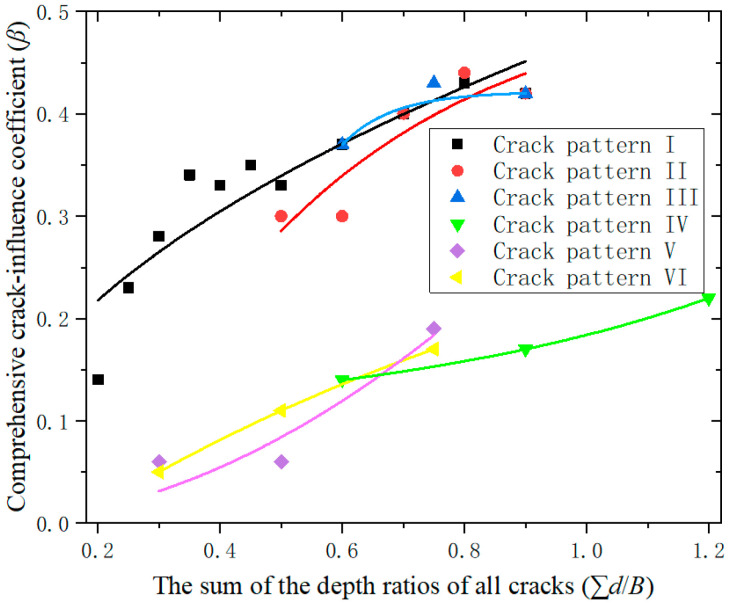
Relationship between the crack influence coefficient and the sum of the crack depth ratio for all six crack-pattern specimens.

Figure 15 compares the comprehensive crack-influence coefficients for specimens under six different crack patterns. It can be observed that the comprehensive crack influence coefficient of crack-pattern-IV, -V, and -VI specimens are significantly lower than that of crack-pattern-I, -II, and -III specimens.

The reason for the above phenomenon is closely related to the location of the cracks. The low comprehensive crack influence coefficient of crack-pattern-IV specimens are due to the minimal impact of two secondary cracks on the load-bearing capacity. The influence of the location of the two secondary cracks on the specimen is not as significant as that of the location of the primary crack.

The low comprehensive crack influence coefficient of crack-pattern-V and -VI specimens are due to the fact that their cracks are located on the top and bottom surfaces. After cracks occur on the top and bottom surfaces, the probability of crack propagation under shear stress is much lower than that of cracks located on the side surfaces. Therefore, at the same crack depth, the impact of cracks on the load-bearing capacity of timber beams on the top and bottom surfaces are much smaller than that of cracks on the side surfaces.

A proper understanding of the above phenomena holds significant guiding value for the assessment and repair of historic timber beams. In actual structures, drying cracks often appear on the side surfaces of beams, particularly at the midpoints of the longer edges of the cross-section, which corresponds precisely to the scenarios simulated by crack patterns -I, -II, and -III in this study. Therefore, during inspection and assessment, priority should be given to evaluating the depth and distribution of cracks on the side surfaces.

### 4.4. Differences from Existing Research and Contributions of This Study

Compared to similar studies, the most prominent contribution of this research is the construction of a complete theoretical framework from crack formation to load-bearing capacity evaluation, achieving the organic integration of wood shrinkage cracking research and wood beam load-bearing capacity research.

Unlike Ivaniuk et al.’s focus on the parameter analysis of cracks penetrating along the neutral axis, this study designed four-point bending specimens with six typical crack distribution patterns, with a focus on revealing the coupling effects of multi-crack systems. In addition, this study also concluded that timber beams with very deep cracks can still maintain 60% of load-bearing capacity, which is different from the conclusion that deep crack causes the timber beam to lose almost all load-bearing capacity.

Compared to the research of Zhang et al., which utilized naturally cracked specimens and DIC technology, this study used small-sized specimens; therefore, it is possible to conduct experiments with large sample sizes, which cannot be achieved by experiments with large-sized specimens. This study quantitatively identified a critical threshold where secondary cracks only begin to significantly impair load-bearing capacity when the main crack depth exceeds 0.45 times the beam width, thereby complementing the theory of crack cluster synergy proposed by Zhang.

Addressing the load-bearing capacity model based on the I-beam assumption established by Bai et al., this study innovatively proposed a “comprehensive crack influence coefficient (*β*)”. This coefficient enables the normalized comparison of cracks with varying depths, numbers, and locations, effectively overcoming the limitation of previous models that could only predict the degradation trend for a single crack.

Regarding the work of Tu et al., who investigated LT-type crack initiation using AE and DIC techniques, this study elevated the findings on microscopic fracture mechanisms to the level of macroscopic structural performance assessment. It provided an in-depth explanation of the mechanical essence behind deeply cracked beams retaining 60% of their load-bearing capacity, thus completing a closed-loop study of crack propagation mechanisms to load-bearing capacity evaluations. By introducing the load-bearing capacity coefficient and the comprehensive influence coefficient, this study offers a concise and comprehensive quantitative tool for the scientific assessment of cracked timber beams in engineering practices.

### 4.5. The Validity and Limitations of This Study

This study adopted a strategy of combining small-sized specimens with a large sample size (*n* = 1200). This approach not only enabled a parameterized experimental data comparison of multiple crack patterns and crack depths but also significantly reduced experimental costs. It is impossible to achieve such a large sample size for testing through full-scale timber beams. Based on the experimental data analysis of this study, a further design of full-scale timber beam experiments can improve the effectiveness of the experiments and reduce wood waste.

In addition, some conclusions of this study have guiding significance for the maintenance of historical buildings: the study reveals that the distribution of cracks has a coupling effect. In actual timber beams, cracks often appear in clusters rather than in isolation. Research has found that when the total crack depth is the same, the decrease in the load-bearing capacity of symmetrical double cracks is slightly lower than that of single cracks, while in multi-crack systems, secondary cracks will interact with the primary crack, jointly weakening the section. This finding suggests that in the assessment of historical timber structures, attention should not be confined solely to the deepest visible crack. Instead, the spatial distribution characteristics and synergistic effects of the entire crack system must be taken into account.

Furthermore, the study indicates that cracks located on the side surfaces of a beam have a far more significant weakening effect on load-bearing capacity than those on the top or bottom surfaces. In actual timber components, drying cracks are most likely to initiate at the midpoint of the long side of the cross-section. Therefore, during on-site inspections, priority should be given to evaluating the depth and morphology of side cracks within limited working hours. Such a focused approach can substantially improve assessment efficiency and avoid unnecessary time expenditure.

This study also has limitations, mainly including “size effect related to small-sized specimens”, “differences between artificial cracks and naturally formed cracks”, “simplified FE material parameters and moisture content conditions”, etc.

The usage of small-sized specimens introduces size effects and material inhomogeneity. Wood, as a naturally anisotropic material, exhibits mechanical properties significantly influenced by its microstructure (e.g., fiber orientation, distribution of earlywood and latewood). The number of annual rings contained in small-sized specimens is less than that of full-size timber beams. The proportion of earlywood and latewood in different small-sized specimens will have greater variability, which makes the experimental results of small-sized specimens more variable than those of full-size specimens.

This study attempted to eliminate the influence of specimen variability as much as possible through a large sample size in experiments. However, this can only improve the accuracy of the experiment statistically; it cannot completely replace an experiment of full-size timber beams. Due to the size effect, the reduction rate of load-bearing capacity obtained from small-sized specimens is not equivalent to that of full-size timber beams. Therefore, the load-bearing capacity data provided in this study are only used to compare the influence of different crack patterns and crack depths on the load-bearing capacity of specimens and cannot be directly used to evaluate the load-bearing capacity of full-size timber beams.

In this study, artificial methods were used to pre-fabricate cracks in specimens, resulting in highly consistent crack shapes (such as 40 samples with identical crack sizes and shapes), which makes the experiment highly reproducible. However, it should be noted that the shape of cracks in real timber beams is complex and the shape and depth of cracks in each beam are different. There may even be phenomena such as crack branching and crack bridging. Therefore, this study can only predict the basic formation rules of possible cracks in timber beams and cannot predict the specific shapes of cracks, nor can it predict the specific depth that a crack can achieve in practice.

In addition, the wood material parameters and drying process-related parameters in the FE model have also been simplified. This is to make the method of this study easy to operate and to increase the reproducibility of simulation results. In fact, the material properties of wood are very complex, not only with different elastic moduli in tension and compression, but also with viscoelastic characteristics, which can affect the load-bearing capacity of real timber beams. The drying processes of real timber beams are also very complex and are not only affected by temperature, air humidity, air circulation speed, etc., but also by the microstructure of wood—such as annual rings. During the drying process, the phenomenon of cupping may even occur. In order to avoid overly complex simulation models, this study only achieved simulation through the simplest geometry and material parameter settings, aiming to reveal the impact of changes in key parameters on the drying and cracking processes of timber beams. However, the specific data in the simulation results (such as drying time, depth of stress region, etc.) cannot represent the situation in real timber beams.

In future work, the research team will consider gradually reducing the sample size while increasing the specimen dimensions, aiming to identify the most suitable specimen size from the perspective of balancing size effects and experimental costs. Although full-scale timber beam tests most closely represent real structural behavior, it is impractical to conduct such tests with sample sizes on the order of 1000. From this standpoint, experiments on small-sized specimens remain both important and necessary.

## 5. Conclusions

This study systematically investigated the influence of six typical surface shrinkage crack distribution patterns (patterns I–VI) on the flexural load-bearing capacity of timber beams under varying crack depths, using systematic four-point bending tests. The experimental results show that crack depth is a key factor affecting load-bearing capacity. When the crack is shallow (*d*/*B* ≤ 0.2), the reduction in the load-bearing capacity of the timber beam is less than 5%, exhibiting a limited impact on structural safety. When the crack depth reaches approximately one-third of the beam width, the reduction in load-bearing capacity increases to around 10%; this depth can serve as an engineering warning threshold. Even with a crack depth of 90% of the beam width, the timber beam retains about 60% of its original load-bearing capacity, indicating that the remaining cross-section can still contribute to loadbearing.

Crack distribution patterns exhibit a noticeable coupling effect. Under the same total crack depth, specimens with symmetric double cracks (pattern II) show a slightly lower reduction in load-bearing capacity compared to those with single cracks (pattern I) and asymmetric double cracks (pattern III), suggesting that a symmetric layout facilitates stress redistribution. In the multi-crack system (pattern IV), when the main crack is relatively deep (*d*/*B* > 0.45), secondary cracks further reduce load-bearing capacity, indicating that the synergistic effects of crack clusters must be considered during assessment.

A crack’s location plays a decisive role in its influence. Cracks located on the side surfaces of timber beams (patterns I–III) more significantly weaken load-bearing capacity more than those on the top or bottom surfaces (patterns V–VI), which is related to the gradient distribution of bending stress along the cross-sectional height and the transition in failure modes. In practical engineering, side-surface cracks—especially those at the midpoints of the longer edges of the cross-section—should be prioritized during inspection and assessment.

By introducing the load-bearing capacity coefficient (*R*), the decline ratio of load-bearing capacity (*D*), and the comprehensive crack-influence coefficient (*β*), this study enabled quantitative comparisons of different crack characteristics, providing a simple tool for assessing the load-bearing capacity of cracked timber beams.

In summary, this study revealed quantitative relationships between the shrinkage crack depth, location, and distribution patterns and the flexural performance of timber beams. It emphasizes the need to consider the overall characteristics of crack systems in practical engineering assessments, rather than solely focusing on the deepest crack. The findings provide theoretical foundations and data support for the damage assessment, repair, and reinforcement of timber members in historic buildings and modern timber structures. Future work may further combine full-scale tests and multi-scale numerical simulations to deepen the understanding of crack interaction mechanisms and size effects.

## Figures and Tables

**Table 1:** Material properties, initial and boundary conditions of timber beam cross-sections in FE simulations.

Model Part	Parameters	Parameters from
Material Directions	1 → R direction, 2 → T direction, 3 → L direction	
Elastic constants	*E*_1_ = 0.8 GPa, *E*_2_ = 0.4 GPa, *E*_3_ = 10.0 GPa, *ν*_12_ = 0.73, *ν*_13_ = 0.052, *ν*_23_ = 0.034, *G*_12_ = 0.07 GPa, *G*_13_ = 0.62 GPa, *G*_23_ = 0.54 GPa	Reference [27]
Ultimate stress	*σ*_T1_ = 3.7 MPa, *σ*_T2_ = 2.1 MPa, *σ*_T3_ = 66.8 MPa	Reference [28]
Moisture diffusion and shrinkage coefficient	*MD*_1_ = 1.8 × 10^−10^ m^2^s^−1^, *MD*_2_ = 1.9 × 10^−10^ m^2^s^−1^, *MD*_3_ = 1.4 × 10^−9^ m^2^s^−1^ *α*_1_ = 0.144, *α*_2_ = 0.324, *α*_3_ = 0.01	References [29,30]
Initial and boundary conditions	*MC*_0_ = 30%, *MC*_B_ = 12% *Φ_v_* = {0.1, 1.0, 10, 100, 1000} × 10^−6^ kg·m^−2^s^−1^	Reference [31]

**Table 2:** The time required for each stage with different *Φ_v_*.

*Φ_v_*/(×10^−6^ kg·m^−2^s^−1^)	Stage 1	Stage 2	Stage 3	Stage 4
0.1	/	/	/	9790 days
1.0	11.6 days	118 days	155 days	1516 days
10	11 h	25 days	90 days	650 days
100	1.0 h	5.1 days	76 days	561 days
1000	0.1 h	4.8 days	72 days	555 days

Note: stage 1 represents the onset of cracks; stage 2 represents the occurrence of maximum stress; stage 3 represents the tensile stress region reach its maximum depth; and stage 4 represents the end of the drying process.

**Table 3:** Crack depth setting for each crack-pattern specimen in the experiments.

Type of Specimen	Crack Depths/(*d*/*B*)
Crack-pattern-I	0.20, 0.25, 0.30, 0.35, 0.40, 0.45, 0.50, 0.60, 0.70, 0.80, 0.90
Crack-pattern-II	0.25, 0.30, 0.35, 0.40, 0.45
Crack-pattern-III	0.40, 0.50, 0.60
Crack-pattern-IV	0.30, 0.45, 0.60
Crack-pattern-V, -VI	0.30, 0.50, 0.75

**Table 4:** Variations in specimen size and weight in 40 specimens.

Parameter	Average	Maximum	Minimum	*SD*	*CV*/%
*L*/(mm)	160.1	161.1	159.1	0.400	0.25
*B*/(mm)	9.9	10.3	9.7	0.171	1.72
*H*/(mm)	15.1	15.2	14.8	0.088	0.58
*m*/(g)	11.4	12.2	10.5	0.419	3.68

**Table 5:** Examples of the fracture load adjustment under three cases.

No.	*m*/(g)	*L*/(mm)	*b*/(mm)	*h*/(mm)	∑*d*/(mm)	*ρ*/(kg·m^−3^)	*C_ρ_*	*C_W_*	*P*/(N)	*P_e_*/(N)
1	10.50	159.9	9.9	14.9	0.0	445	1.079	1.024	1206	1333
2	11.30	160.0	10.0	15.0	3.0	476	1.008	1.000	1377	1388
3	12.15	160.1	10.1	15.2	12.0	514	0.934	0.964	1502	1352

**Table 6:** Typical data dispersion levels in the research, corresponding *CV*s, and sample sizes required in experiments.

Data Dispersion Level	Typical *CV*	Sample Size (Error Proportion = ±10%)	Sample Size (Error Proportion = ±7.5%)	Sample Size (Error Proportion = ±5%)
Low	10.9%	5	11	19
Medium	15.1%	9	20	35
High	19.7%	15	34	60

**Table 7:** Experimental data of crack-pattern-I specimens under various crack depth ratios.

*d*/*B*	Sample Size	Equivalent Fracture Load			*R* ± 95%*CI*	*D/%* ± 95%*CI*	*β* ± 95%*CI*	*Tukey HSD α* = 0.05
		Average, Max, Min/(N)	*SD*/(N)	*CV*/%				
0.20	40	1433, 2014, 924	239	16.7	0.973 ± 0.050	2.8 ± 5.0	0.14 ± 0.25	A
0.25	40	1391, 1774, 1045	200	14.4	0.944 ± 0.042	5.7 ± 4.2	0.23 ± 0.16	AB
0.30	40	1330, 1809, 980	177	13.3	0.903 ± 0.037	9.7 ± 3.7	0.32 ± 0.12	ABC
0.35	40	1296, 1623, 1000	178	13.7	0.880 ± 0.037	12.0 ± 3.7	0.34 ± 0.11	BC
0.40	40	1281, 1946, 834	209	16.3	0.870 ± 0.044	13.0 ± 4.4	0.33 ± 0.11	BC
0.45	40	1244, 1573, 953	164	13.2	0.844 ± 0.035	15.6 ± 3.5	0.35 ± 0.08	CD
0.50	40	1229, 1602, 921	149	12.1	0.834 ± 0.031	16.6 ± 3.1	0.33 ± 0.06	CD
0.60	40	1151, 1424, 845	125	10.9	0.781 ± 0.026	21.9 ± 2.6	0.37 ± 0.04	DE
0.70	40	1062, 1428, 804	151	14.2	0.721 ± 0.032	27.9 ± 3.2	0.40 ± 0.05	EF
0.80	40	963, 1431, 680	164	17.0	0.654 ± 0.034	34.6 ± 3.4	0.43 ± 0.04	FG
0.90	40	916, 1184, 727	159	17.4	0.622 ± 0.034	37.8 ± 3.4	0.42 ± 0.04	G

Note: A, B, C, D, E, F, G represent the groups, with no significant differences between groups of the same letter but significant differences between groups of different letters. For *R*, *D* and *β* values, the error range under 95% confidence interval conditions is provided, the meaning of *SD* in the table is standard deviation, the meaning of *CV* is coefficient of variation. The subsequent data tables are also the same as this table.

**Table 8:** Experimental data of crack-pattern-II and -III specimens under various crack depth ratios.

Depth Ratio of Crack-1, -2	Sample Size	Equivalent Fracture Load			*R* ± 95%*CI*	*D/%* ± 95%*CI*	*β* ± 95%*CI*
		Average, Max, Min/(N)	*SD*/(N)	*CV*/%			
0.25, 0.25	40	1247, 1539, 963	145	11.6	0.850 ± 0.030	15.0 ± 3.0	0.30 ± 0.06
0.30, 0.30	40	1207, 1535, 898	142	11.8	0.819 ± 0.030	18.1 ± 3.0	0.30 ± 0.05
0.40, 0.20	40	1143, 1529, 686	157	13.7	0.776 ± 0.033	22.4 ± 3.3	0.37 ± 0.06
0.35, 0.35	40	1055, 1484, 730	142	13.5	0.716 ± 0.030	28.4 ± 3.0	0.40 ± 0.04
0.50, 0.25	40	998, 1269, 653	144	14.4	0.678 ± 0.030	32.2 ± 3.0	0.43 ± 0.04
0.40, 0.40	40	955, 1293, 636	172	18.0	0.649 ± 0.037	35.1 ± 3.7	0.44 ± 0.05
0.45, 0.45	40	910, 1312, 635	144	15.8	0.618 ± 0.030	38.2 ± 3.0	0.42 ± 0.03
0.60, 0.30	40	918, 1220, 484	181	19.7	0.623 ± 0.038	37.7 ± 3.8	0.42 ± 0.04

**Table 9:** Experimental data of crack-pattern-IV specimens under various crack depth ratios.

Depth Ratio of Crack-1, -2, -3	Sample Size	Equivalent Fracture Load			*R* ± 95%*CI*	*D/%* ± 95%*CI*	*β* ± 95%*CI*
		Average, Max, Min/(N)	*SD*/(N)	*CV*/%			
0.30, 0.15, 0.15	40	1349, 2244, 1044	219	16.2	0.915 ± 0.046	8.5 ± 4.6	0.14 ± 0.08
0.45, 0.225, 0.225	40	1246, 1525, 922	142	11.4	0.845 ± 0.030	15.5 ± 3.0	0.17 ± 0.03
0.60, 0.30, 0.30	40	1087, 1503, 876	132	12.1	0.737 ± 0.028	26.3 ± 2.8	0.22 ± 0.02

**Table 10:** Experimental data of crack-pattern-V and -VI specimens under various crack depth ratios.

*d*/*B*	Crack Pattern	Sample Size	Equivalent Fracture Load			*R* ± 95%*CI*	*D/%* ± 95%*CI*	*β* ± 95%*CI*
			Average, Max, Min/(N)	*SD*/(N)	*CV*/%			
0.30	V	40	1448, 2172, 1036	256	17.7	0.983 ± 0.054	1.7 ± 5.4	0.06 ± 0.18
	VI	40	1451, 2264, 930	250	17.2	0.985 ± 0.053	1.5 ± 5.3	0.05 ± 0.18
0.50	V	40	1426, 2021, 942	220	15.4	0.968 ± 0.046	3.2 ± 4.6	0.06 ± 0.09
	VI	40	1389, 1923, 713	240	17.3	0.943 ± 0.051	5.7 ± 5.1	0.11 ± 0.10
0.75	V	40	1268, 1719, 910	191	15.1	0.861 ± 0.040	13.9 ± 4.0	0.19 ± 0.05
	VI	40	1287, 1671, 831	216	16.8	0.874 ± 0.046	12.6 ± 4.6	0.17 ± 0.06

**Table 11:** The ratio of the maximum stress in the T direction in crack-pattern-I, -II, and -III specimens.

Σ*d*/*B*	Crack-Pattern-I Specimen		Crack-Pattern-II-Specimen		Crack-Pattern-III-Specimen	
	Tension	Compression	Tension	Compression	Tension	Compression
0.3	0.3	2.3	0	1	0	1.4
0.6	3.5	5.5	0	1	0.5	2.5
0.9	26	28	0	1	8	10

## Data Availability

The original contributions presented in this study are included in the article. Further inquiries can be directed to the corresponding author.

## Abbreviations

The following abbreviations are used in this manuscript:

CI	Confidence interval
CV	Coefficient of variation
EF	Evaporation flux
FE	Finite element
MC	Moisture content
MD	Moisture diffusion coefficient
SD	Standard deviation
